# Supplementary material for: Serum Metabolomic Profiling of Patients with Non-Infectious Uveitis
Source: J Clin Med. 2020 Dec 6;9(12):3955. doi: 10.3390/jcm9123955 (PMC7762156; doi:10.3390/jcm9123955)
Supplement: Supplementary file 1 [file jcm-09-03955-s001.zip › Supplement Files/Supplement Figure 1.pdf]

Supplement Figure 1

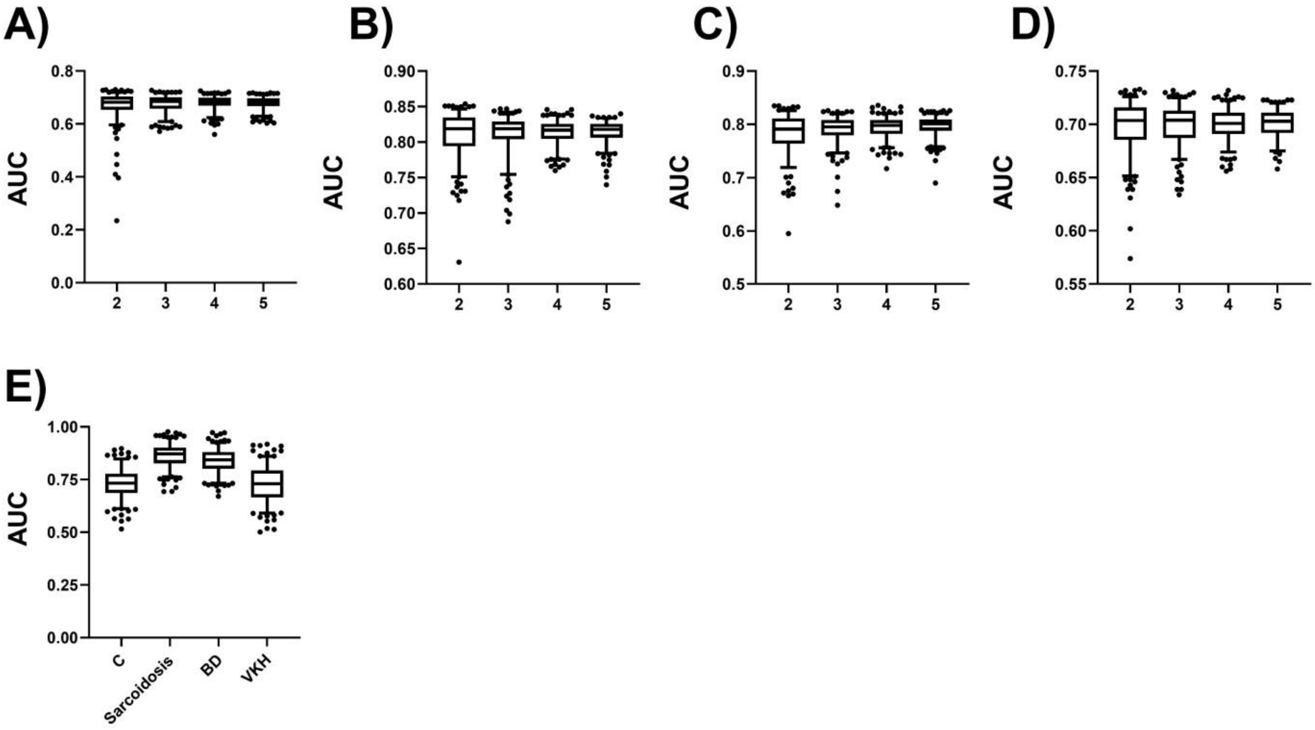

Supplement Figure 1.
Distribution of AUC values yielded by CV and resampling test.
(A)-(D) AUC values of CV for the comparisons of (a)-(d) in Figure 1. The X-axis indicates the k-values of CV. (E) AUC values of resampling tests for the comparisons of (a)-(d) in Figure 1.
The horizontal bars of box plots indicate 5%, 25%, 50%, 75%, and 95%, and the data <5% and >95% are shown in dots.
