# Supplementary material for: Serum Metabolomic Profiling of Patients with Non-Infectious Uveitis
Source: J Clin Med. 2020 Dec 6;9(12):3955. doi: 10.3390/jcm9123955 (PMC7762156; doi:10.3390/jcm9123955)
Supplement: Supplementary file 1 [file jcm-09-03955-s001.zip › Supplement Files/Supplement Table 1.pdf]

Supplementary table 1. LC gradients for negative mode

Time	Mobile phase	
(min)	A (%)	B (%)
0.00	100	0
1.00	100	0
1.01	95	5
1.50	95	5
1.51	70	30
2.50	70	30
2.51	40	60
3.50	40	60
3.51	15	85
4.00	15	85
4.01	5	95
6.00	5	95
