# Supplementary material for: Serum Metabolomic Profiling of Patients with Non-Infectious Uveitis
Source: J Clin Med. 2020 Dec 6;9(12):3955. doi: 10.3390/jcm9123955 (PMC7762156; doi:10.3390/jcm9123955)
Supplement: Supplementary file 1 [file jcm-09-03955-s001.zip › Supplement Files/Supplement Table 2.pdf]

Supplementary table2: List of quantified metabolites

Compound name	Formula	KEGG ID	HMDB ID	HMDB Class	m/z	Retention time(min)
1-Methyladenosine	C11H15N5O4	C02494	HMDB0003331	Purine nucleosides	282.1197	3.131
1-Methylnicotinamide	C7H9N2O	C02918	HMDB0000699	Pyridines and derivatives	137.0709	1.006
2-Aminoadipate	C6H11NO4	C00956	HMDB0000510	Carboxylic acids and derivatives	162.0761	0.926
2-Hydroxyglutarate	C5H8O5	C02630	HMDB0059655	Hydroxy acids and derivatives	147.0298968	0.715
2-Oxoglutarate	C5H6O5	C00026	HMDB0000208	Keto acids and derivatives	145.0142468	0.682
3-Methyl-2-oxobutanoate	C5H8O3	C00141	HMDB0000019	-	115.0400676	1.792
3-Methylhistidine	C7H11N3O2	C01152	HMDB0000479	Carboxylic acids and derivatives	170.0924	1.072
4-Guanidinobutanoate	C5H11N3O2	C01035	HMDB0003464	Carboxylic acids and derivatives	146.0924	2.548
7-Methylguanine	C6H7N5O	C02242	HMDB0000897	Imidazopyrimidines	166.0723	2.498
Adenosine	C10H13N5O4	C00212	HMDB0000050	Purine nucleosides	268.104	3.313
ADMA	C8H18N4O2	C03626	HMDB0001539	Carboxylic acids and derivatives	203.1503	2.548
Alanine	C3H7NO2	C00041	HMDB0000161	Carboxylic acids and derivatives	90.055	0.724
AMP	C10H14N5O7P	C00020	HMDB0000045	-	346.0558078	0.665
Arginine	C6H14N4O2	C00062	HMDB0000517	Carboxylic acids and derivatives	175.119	1.34
Asparagine	C4H8N2O3	C00152	HMDB0000168	Carboxylic acids and derivatives	133.0608	0.608
Aspartate	C4H7NO4	C00049	HMDB0000191	Carboxylic acids and derivatives	134.0448	0.608
beta-Alanine	C3H7NO2	C00099	HMDB0000056	Carboxylic acids and derivatives	90.055	0.909
Carnitine	C7H15NO3	C00318	HMDB0000062	Organonitrogen compounds	162.1125	1.006
Choline	C5H14NO	C00114	HMDB0000097	-	104.107	0.956
Citrate	C6H8O7	C00158	HMDB0000094	Carboxylic acids and derivatives	191.0197261	0.748
Citrulline	C6H13N3O3	C00327	HMDB0000904	Carboxylic acids and derivatives	176.103	0.708
Creatine	C4H9N3O2	C00300	HMDB0000064	-	132.0768	0.956
Creatinine	C4H7N3O	C00791	HMDB0000562	Carboxylic acids and derivatives	114.0662	1.122
Cystathionine	C7H14N2O4S	C02291	HMDB0000099	-	223.0747	0.691
Cysteine	C3H7NO2S	C00097	HMDB0000574	Carboxylic acids and derivatives	122.027	0.675
Diethanolamine	C4H11NO2	C06772	HMDB0004437	Organonitrogen compounds	106.0863	0.923
Fumarate	C4H4O4	C00122	HMDB0000134	Carboxylic acids and derivatives	115.0036821	0.649
GABA	C4H9NO2	C00334	HMDB0000112	-	104.0706	1.072
gamma-Butyrobetaine	C7H16NO2	C01181	HMDB0001161	Fatty Acyls	146.1176	1.188
Gluconate	C6H12O7	C00257	HMDB0000625	Organooxygen compounds	195.0510262	0.5
Glutamate	C5H9NO4	C00025	HMDB0000148	Carboxylic acids and derivatives	148.0604	0.691
Glutamine	C5H10N2O3	C00064	HMDB0000641	Carboxylic acids and derivatives	147.0764	0.642
Glycine	C2H5NO2	C00037	HMDB0000123	-	76.0393	0.658
Glycolate	C2H4O3	C00160	HMDB0000115	Hydroxy acids and derivatives	75.00876746	0.49975
Glyoxylate	C2H2O3	C00048	HMDB0000119	-	72.9931174	0.5
Guanosine	C10H13N5O5	C00387	HMDB0000133	-	284.0989	2.415
Hexylamine	C6H15N	C08306	HMDB0032323	Amines	102.1277	5.531
Histidine	C6H9N3O2	C00135	HMDB0000177	Carboxylic acids and derivatives	156.0768	1.023
Homoserine	C4H9NO3	C00263	HMDB0000719	Carboxylic acids and derivatives	120.0655	0.675
Hydroxyproline	C5H9NO3	C01157	HMDB0000725	Carboxylic acids and derivatives	132.0655	0.592
Indoleacetate	C10H9NO2	C00954	HMDB0000197	Indoles and derivatives	176.0706	5.332
Inosine	C10H12N4O5	C00294	HMDB0000195	Purine nucleosides	269.0881	1.52
Isoleucine	C6H13NO2	C00407	HMDB0000172	Carboxylic acids and derivatives	132.1019	3.956
Lactate	C3H6O3	C00186	HMDB0000190	Hydroxy acids and derivatives	89.02441753	0.699
Leucine	C6H13NO2	C00123	HMDB0000687	Carboxylic acids and derivatives	132.1019	4.089
Lysine	C6H14N2O2	C00047	HMDB0000182	Carboxylic acids and derivatives	147.1128	1.175
Malate	C4H6O5	C00711	HMDB0000744	Hydroxy acids and derivatives	133.0142468	0.649
Methionine	C5H11NO2S	C00073	HMDB0000696	Carboxylic acids and derivatives	150.0583	2.067
N1,N8-Diacetylspermidine	C11H23N3O2	-	HMDB0041947	Carboxylic acids and derivatives	230.1863	3.658
N1-Acetylspermidine	C9H21N3O	C00612	HMDB0001276	Carboximidic acids and derivatives	188.1757	3.94
N1-Acetylspermine	C12H28N4O	C02567	HMDB0001186	Carboxylic acids and derivatives	245.2336	5.017
N6,N6,N6-Trimethyllysine	C9H20N2O2	C03793	HMDB0001325	Carboxylic acids and derivatives	189.1598	1.205
N8-Acetylspermidine	C9H21N3O	C01029	HMDB0002189	Carboximidic acids and derivatives	188.1757	4.158
N-Acetylglucosamine	C8H15NO6	C00140	HMDB0000215	Organooxygen compounds	244.0792	0.542
N-Acetylputrescine	C6H14N2O	C02714	HMDB0002064	Carboximidic acids and derivatives	131.1179	1.702
N-Epsilon-Acetyllysine	C8H16N2O3	C02727	HMDB0000206	Carboxylic acids and derivatives	189.1234	1.437
Nicotinamide	C6H6N2O	C00153	HMDB0001406	Pyridines and derivatives	123.0553	1.205
O-Acetylcarnitine	C9H18NO4	C02571	HMDB0000201	Fatty Acyls	204.123	2.763
Ornithine	C5H12N2O2	C00077	HMDB0000214	Carboxylic acids and derivatives	133.0972	1.023
Phenylalanine	C9H11NO2	C00079	HMDB0000159	Carboxylic acids and derivatives	166.0863	4.338
Pipecolate	C6H11NO2	C00408	HMDB0000716	Carboxylic acids and derivatives	130.0863	1.404
Proline	C5H9NO2	C00148	HMDB0000162	Carboxylic acids and derivatives	116.0706	0.741
Pyruvate	C3H4O3	C00022	HMDB0000243	Keto acids and derivatives	87.00876746	0.616
SDMA	C8H18N4O2	-	HMDB0003334	Carboxylic acids and derivatives	203.1503	2.746
Serine	C3H7NO3	C00065	HMDB0000187	Carboxylic acids and derivatives	106.0499	0.625
Spermidine	C7H19N3	C00315	HMDB0001257	Organonitrogen compounds	146.1652	4.752
Spermine	C10H26N4	C00750	HMDB0001256	Organonitrogen compounds	203.223	5.481
Succinate	C4H6O4	C00042	HMDB0000254	Carboxylic acids and derivatives	117.0193321	0.996766667
Taurine	C2H7NO3S	C00245	HMDB0000251	Organic sulfonic acids and derivatives	126.0219	0.509
Thiamine	C12H17N4OS	C00378	HMDB0000235	Diazines	265.1118	4.122
Thymine	C5H6N2O2	C00178	HMDB0000262	Diazines	127.0502	1.172
Trimethylamine N-oxide	C3H9NO	C01104	HMDB0000925	Organonitrogen compounds	76.0757	1.122
Tryptophan	C11H12N2O2	C00078	HMDB0000929	Indoles and derivatives	205.0972	4.835
Tyrosine	C9H11NO3	C00082	HMDB0000158	Carboxylic acids and derivatives	182.0812	3.409
Urea	CH4N2O	C00086	HMDB0000294	Organic carbonic acids and derivatives	61.0396	0.528
Uridine	C9H12N2O6	C00299	HMDB0000296	Pyrimidine nucleosides	267.0588	0.874
Urocanate	C6H6N2O2	C00785	HMDB0000301	Azoles	139.0502	2.564
Valine	C5H11NO2	C00183	HMDB0000883	Carboxylic acids and derivatives	118.0863	1.885
